# Vancomycin drug resistance, an emerging threat to animal and public health

**DOI:** 10.3389/fvets.2022.1010728

**Published:** 2022-10-28

**Authors:** Amjad Islam Aqib, Abdullah F. Alsayeqh

**Affiliations:** ^1^Department of Medicine, Cholistan University of Veterinary and Animal Sciences, Bahawalpur, Pakistan; ^2^Department of Veterinary Medicine, College of Agriculture and Veterinary Medicine, Qassim University, Buraidah, Qassim, Saudi Arabia

**Keywords:** food safety, vancomycin, resistance genes, bacteria, gram-positive, gram-negative

## Abstract

The need to supply quality food for the growing human population has led to the revolutionization of food production and processing in recent years. Meanwhile, food production sources are at risk of microbial attack, while the use of antibiotics to counter them is posing another threat to food safety and security. Vancomycin was used as the first line of defense against multiple drug-resistant bacteria salient of which is methicillin-resistant *S. aureus*. The emergence of the vancomycin resistance gene in bacteria impairs the efficacy of antibiotics on the one hand while its harmful residues impart food safety concerns on the other. Currently, a novel set of resistance genes “Van cluster” is circulating in a wider range of bacteria. Considerable economic losses in terms of low production and food safety are associated with this emerging resistance. The current review focuses on the emergence of vancomycin resistance and its impact on food safety. The review proposes the need for further research on the probable routes, mechanisms, and implications of vancomycin resistance from animals to humans and vice versa.

## Introduction

The use of antimicrobials poses a significant threat to animal production and profitability. Although there are Maximum Residue Limits (MRLs) of the approved veterinary drugs for their use to improve animal health, their implementation requires stern legislation and subsequent actions. On the other hand, there are a variety of guidelines in different countries but some of the countries still need to develop these guidelines (1). Implementing a withdrawal period of antibiotics in animal food products is an additional requirement to avoid food safety concerns. At the farm level, withdrawal periods are kept in line with the expected time of consumption of food. It is commonly required that drug residues must be below MRLs. Extra label utilization, miscalculated doses, non-specific use, and failure in observing the prescribed course of antibiotics may result in the spread of antimicrobial resistance.

Antimicrobial consumption through food or any other sources continuously leads to the suppression of intestinal microbiota that guards against pathogenic bacteria. Moreover, their presence in the bloodstream leads to the malfunctioning of various organs (2, 3). Extensive use of antimicrobials in animals may lead to the production of novel resistant bacteria in the environment. Some of the salient bacteria responsible for food safety and security include *Salmonella, E. coli*, and *S. aureus* isolated from various sources in humans and poultry (4–7), dairy milk (8–11), and farm animals' skin and nares (12, 13). The bacteria are developing resistance to multiple antibiotics and thus becoming a challenge to treating an infection with commonly used antibiotics. Among such antibiotics is vancomycin, a member of glycopeptides, and was the primary applied drug of choice against methicillin-resistant *S. aureus* (14). Recent studies showed *S. aureus* is a resistant pathogen against vancomycin (15, 16) thus increasing the burden of antimicrobial drug resistance.

Resistance against vancomycin is found to be due to a cluster of genes known as the “Van cluster” that is found in a number of bacteria such as S. *aureus, E. fecalis*, Actinomycetes spp., *E. faceium, Clostridium difficile*, and anaerobic bacteria. It is pertinent to note that *Enterococcus* spp. appeared to be a prominent reservoir of vancomycin resistance and its spread to other bacteria is horizontal using the conjugation method. It has been found that *Enterococci* contain Inc18 pSK41-like multiple drug-resistant conjugated plasmids while their presence in *S. aureus* has yet not been documented. The transfer to the latter is found to be by plasmids from *E. faecalis* to other bacteria. Worsening of this resistance is thought to be controlled by manipulation of Van cluster from the bacteria. Hence, it provides a better target for new drug development e.g., phosphinate, phosphonate, and hydroxyethylamines can inhibit Van clusters. These inhibitors are mostly used with vancomycin for the effective uptake of antibiotics inside the bacterial cell (17). Such discoveries are necessary for better control of vancomycin resistance. Vancomycin-resistant genes are extensively transferred from animal to human, animal to bacteria, human to bacteria, and bacteria to bacteria. There is a dire need to focus on novel genes in bacteria to address their resistance and impact on animal and public health. The current review thus focuses on vancomycin resistance in bacteria, its mode of spread, and its impact on food safety/security.

## Background of vancomycin

Vancomycin was isolated by E.C Kornfield in year 1957 from *Streptomyces orientalis*, a fungus, located in the deep jungles of Borneo (18). The obtained substance was named “compound 05865” having activity against gram-positive as well as anaerobic bacteria in initial findings. Vancomycin is a tricyclic glycopeptide that acts as a bactericidal against the polymerization of peptidoglycans in the bacterial cell wall. This antibiotic is equally effective against a wider range of pathogens and in surgical procedures to avoid secondary infection. The FDA has approved the use of this drug against *Clostridium difficile, Staphylococcus enterocolitis, Pseudomembranous colitis*, Enterococcal, Staphylococcal, and Streptococcal spp, which are responsible for diarrheas, endocarditis, sepsis, soft skin infection, bone infections, lower respiratory tract infections, and colitis (19). In other studies, there was an emergence of *mecA* or *mecC* gene in *S. aureus* that codes enzymes to crosslink the peptidoglycans in bacterial cell walls which is now considered a potential threat to control *S. aureus*. The enzymes had low affinity for β-lactams and hence there was a development of resistance. The resistance of Staphylococci against antibiotics was on the rise when this drug was developed therefore the FDA approved vancomycin with number 05865 in 1958 to control infection of Staphylococci. In such a scenario, there was a concern that this product would maintain its efficacy/activity or face resistance. A series of *in-vitro* and lab animal experiments were conducted to rule out this concern (20). The off-label uses of this drug consist of catheter-related infections, neonatal prophylaxis, bacterial meningitis, peritonitis, and many more (21). More than half of regularly used antibacterial medications are no longer effective against Gram-negative Pathogens (GNPs) including *E. coli* and *K. pneumoniae*, according to the WHO Global Report on Surveillance of Antimicrobial Resistance (22–24). It is important to note that drugs like colistin, which were used as a last resort, are no more effective against a GNP such as New Delhi metallo-lactamase-1.

## Development of resistance against vancomycin

**Figure 1:**
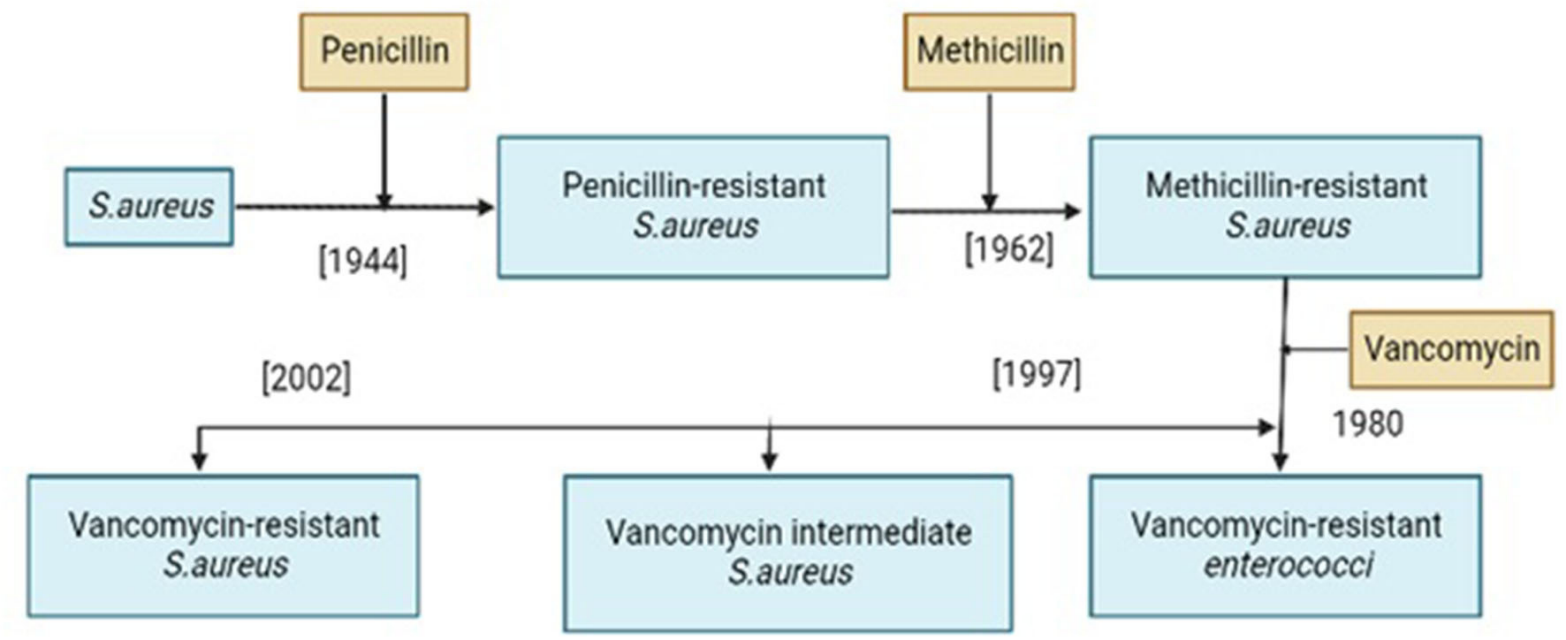
History of resistance development in *Staphylococcus aureus* against different antibiotics.

**Figure 2:**
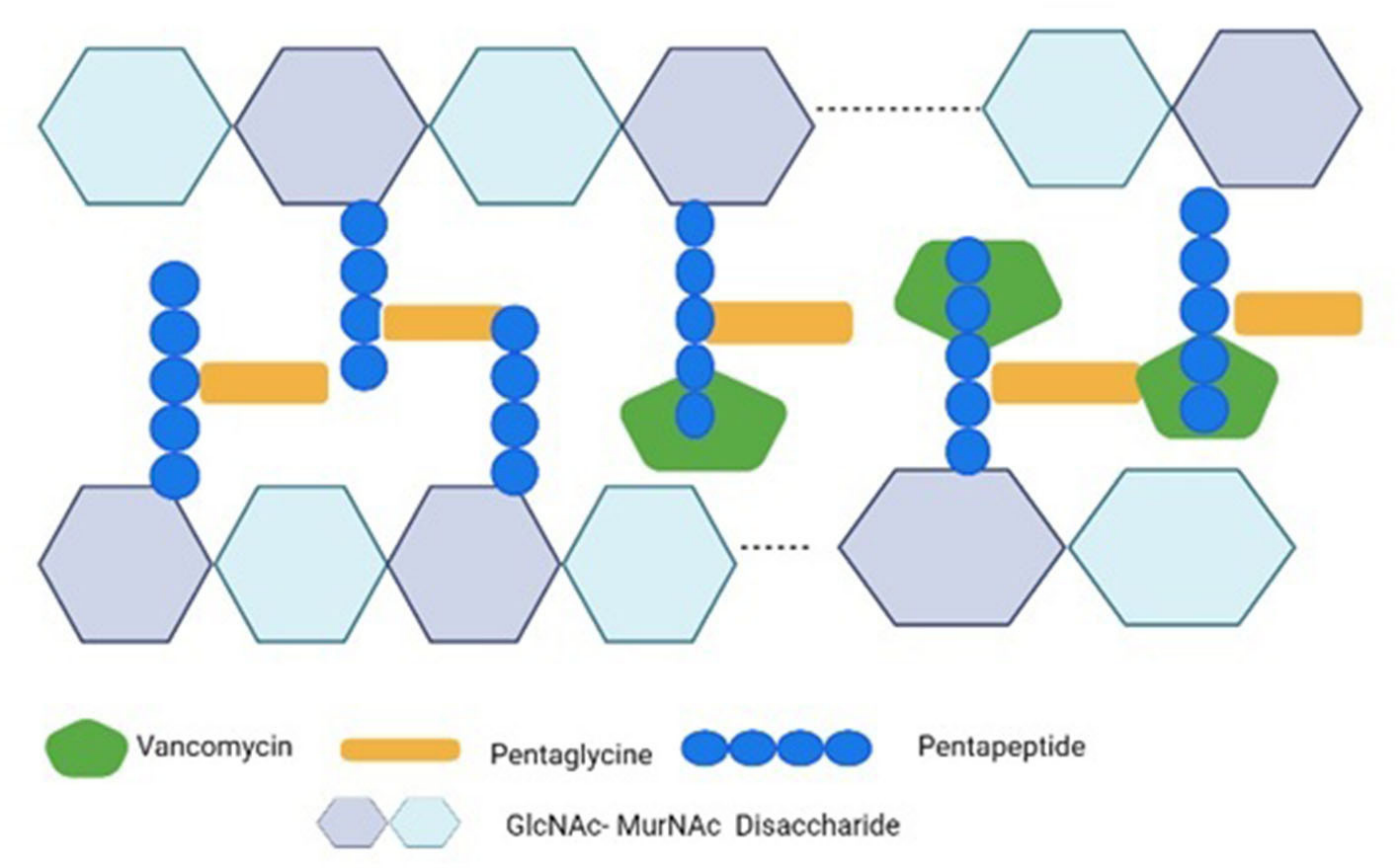
Vancomycin's mechanism of action against S. aureus. Vancomycin inhibits the cross-bridge formation between pentagen and pentaglycine by binding to the D-Ala-D-Ala residues of pentapeptide's C-terminal D-Ala-D-Ala. The GlcNAc stands for N-acetylglucosamine; the MurNAc stands for N-acetylmuramic acid.

Vancomycin resistance was first reported in *Enterococcus faecium* in 1986 (25) and the resistant genes were named VRE (Vancomycin-resistant *Enterococci*). Avoparcin was overused as a growth enhancer in animal production in European communities. In addition to *Enterococci*, there was also a development of novel strains of *S. aureus* against vancomycin (Figures 1, 2) that gave rise to three distinct categories (i) VSSA (*S. aureus* sensitive to vancomycin), (ii) VRSA (*S. aureus* resistant to vancomycin), and (iii) VISA (*S. aureus* intermediate susceptible to vancomycin).

### Acquired resistance

**Figure 3:**
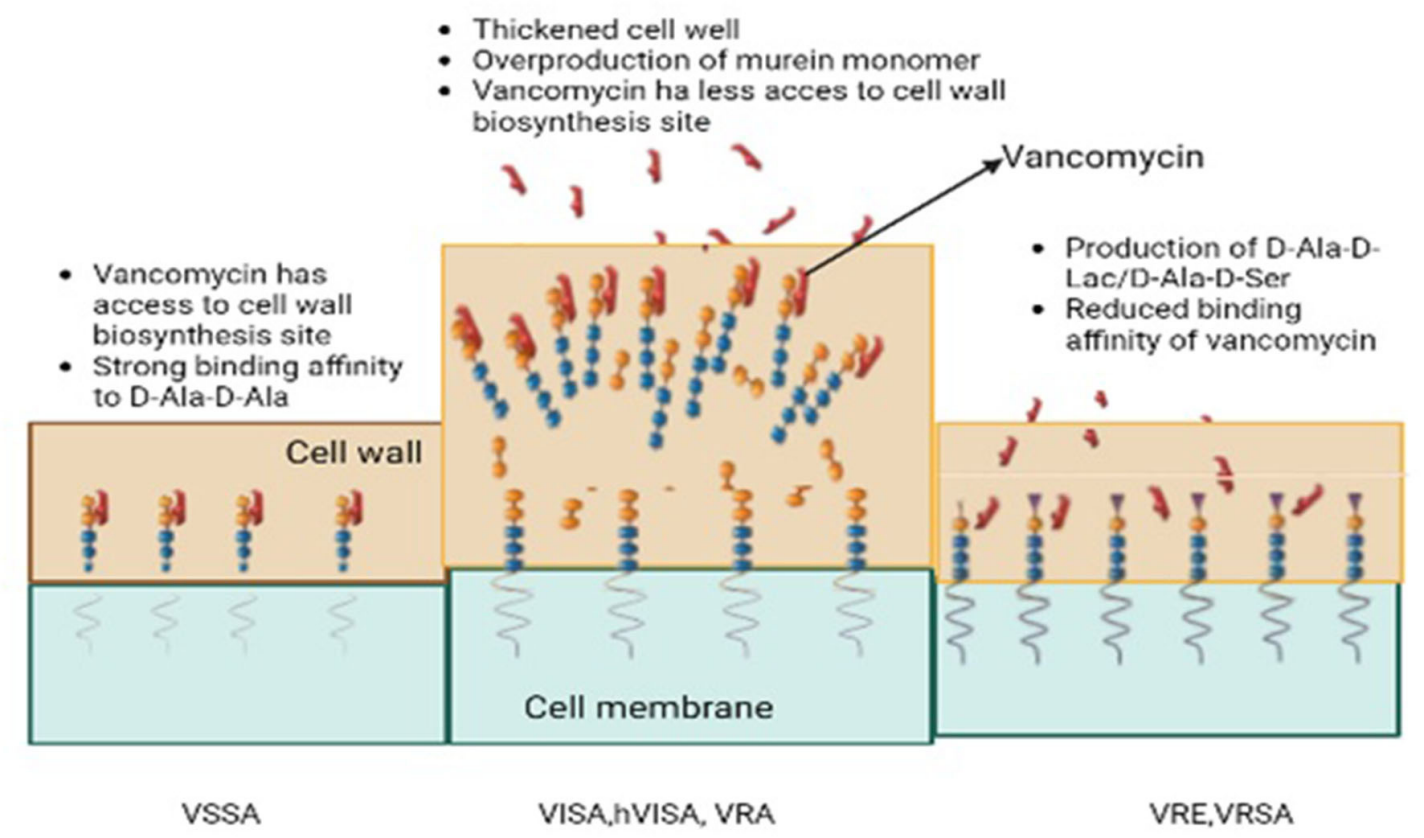
The development of acquired resistance; Vancomycin-resistant S. aureus (VRSA), Vancomycin-resistant Enterococci (VRE), Vancomycin susceptible S. aureus, Vancomycin intermediate S. aureus (VISA), Hetero-vancomycin intermediate S. aureus (hVISA).

This kind of resistance resulted in a synthesis of precursors of peptidoglycan which ends in D-Ala-D-Lac and D-Ala-D-Ser instead of D-Ala-D-Ala (Figure 3). These precursors are highly incompatible with vancomycin and thus give rise to a cluster of 11 genes found primarily in Enterococci to confer vancomycin resistance. The precursor ending with D-Ala-D-Lac is coded by vanA, B, D, F, I, and M clusters while that of D-Ala-DSer is coded by vanC, E, G, L, and N clusters. It was found that the vanA gene and vanB gene are frequently encountered genes when it comes to acquired resistance (26, 27). The ability of vancomycin to bind to targeted peptides is 1000 times less when these peptidoglycan precursors are produced with an association constant of 102 M-1 (28).

The gene clusters vanA and vanB consist of vanX, vanH, vanA, and other related homologous variant genes (29). Activating transcription and regulating cytoplasmic responses is achieved by phosphorylating the vanR protein. Among them are vanR, which upregulates the expression of vanR & S genes, and vanHAX. They bind to the core of vanHAX and vanH which encodes a D-lactate dehydrogenase converting pyruvate into D-lactate and vanX. Finally, the products (D-lactate and vanX) encode a D, D-dipeptidase that further cleaves D-Ala–D-Ala (30). The vanA and B clusters also contain vanY that encodes a D, D-carboxypeptidase. A late membrane-bound peptidoglycan precursor is cleaved at the C-terminus by this enzyme. Vancomycin intermediate susceptible *S. aureus* (VISA) also contains mutations in the RNA polymerase gene rpoB which regulates transcription of genes in cell wall biosynthesis but mutated genes in the walkR system (walkR system, yvqF/vraSR system) seem to be directly or indirectly related to the biosynthesis or metabolism of those two-component sensory regulatory systems (31). In addition to this, lactamase-encoding genes have also exhibited contributions to the expression of VISA (32). VISA isolates demonstrate several common characteristics such as excessive quantities in the cell wall, abnormal daughter cells, post-cellular division, and reduced rates of autolysis. The altered phenomena behind these molecular mechanisms are yet to be understood due to abnormally high amounts of synthesized peptidoglycan. The activation of the arginine catabolism pathway (*via* an activated ADI pathway) in VISA strains (Mu50 lineage) has been suggested to provide ATP molecules and ammonia to compensate for increased cell wall biosynthesis. There is a need for further research to investigate this phenomenon globally in different strains of VISA. There was also a decrease in the cross-linking of peptidoglycan strands in the thickened wall, leading to an increase in non-cross-linked D-alanyl-D-alanine side chains. In addition to the binding of vancomycin to DAla-D-Ala, the bound vancomycin also serves as an obstacle to the free molecules found deeper within the structure (33).

### Intrinsic resistance

The gram-negative bacteria had an additional outer membrane that acted as a barrier for the entry of glycopeptides-like hydrophilic molecules. The higher molecular weight and size of these molecules prevent them from entering through the porins of the outer membrane to reach the target site within the cell wall region. This mechanism results in resistance of gram-negative bacteria against glycopeptides and is called ‘intrinsic resistance'. The main contributor to this intrinsic resistance appears to be an extra outer membrane (OM) and numerous efflux pumps which successfully block the entry of several pharmacological compounds including glycopeptide antibiotics like vancomycin (34). According to Hancock and Brinkman (35) and Pagès et al. (36) hydrophobic medicines passively pass through the OM whereas hydrophilic antibiotics diffuse through porin channels. Glycopeptides have a complicated chemical structure, a large molecular weight, and are hydrophilic by nature (1,450–2,000 Da). They are hydrophilic but because of their enormous size and molecular weight, they cannot pass through the OM's porins to the cell wall region. GNPs inherently resist glycopeptides because they operate within cell walls.

### Role of avoparcin in resistance development

Diseases were non-responsive to the antibiotics intended to be used as growth promotors for better FCR (feed conversion ratio) and reduced morbidity/mortality amid clinical/subclinical infections (37). Avoparcin was mainly used to treat animals including broilers, turkeys, pigs, and veal calves (38). Data from Denmark shows the breadth of avoparcin use where >24,000 kg was used for growth promotion whereas 24 kg of the drug was utilized to treat humans. In Australia, <600 kg of vancomycin and nearly 62,000 kg of avoparcin were purchased during 1992–996 in similar circumstances. Avoparcin provides cross-resistance to vancomycin, hence it is improperly used to treat VRE (38). The most clinically significant species is *Enterococcus faecalis* and the strains from animals may pose a risk to humans. For instance, it is believed that human VRE outbreaks in some countries were influenced by the rise of vancomycin-resistant enterococci (VRE). This factor is attributed to excessive usage of the vancomycin derivative avoparcin as a growth promoter in farm animals (39). Until it was forbidden in 1997, the widespread use of avoparcin as a growth enhancer in the agricultural sector was linked to the frequent isolation of VRE from farm animals (40). The first antibiotic feed additive used in food-producing animals banned in 1997 by the EU was avoparcin. This antibiotic was banned from use in food-producing animals because it belongs to the glycopeptide class, a critically important antibiotic used in human medicine. Resistant enterococci have also been isolated from the raw meat of animals fed avoparcin, creating a concern for the passage of resistant enterococci to people *via* the food chain (41). This is what led to the ban of avoparcin as an antibiotic feed additive in the EU.

### Exploring resistance in different classes of bacteria

During the 1990s*, E. faecium* with the vanA genotype, or VRE, was prevalent in the intestinal flora of farmed animals in Europe (42, 43). There are several explanations for why VRE still exists in livestock animals. Because the genes for both resistances were found on the same plasmid, a study in Denmark suggested the use of a combination of macrolide and tylosin as the solution for VRE (44). In another study, bacteria are forced to maintain resistance if plasmid addiction systems were present on the same plasmid as the vanA gene (45). The three most prevalent types are vanA, vanB, and vanC, and among these the vanA genotype is considered the most abundant (46, 47). A second variety (vanF) has also been discovered even though it has only been found in *Paenibacillus popilliae* thus far. *P. popilliae* has been proposed as a potential source of vancomycin resistance in enterococci due to a considerable degree of similarity between amino acid sequences of the vanF variant and vanA variant (48). Other potential origins include a variety of organisms that produce glycopeptides even if there is a more likely development of genetic variances as an earlier common source. The initial D-Ala-D-Ala pentapeptide found within the three-dimensional network of peptidoglycans that makes up the bacterial cell wall may be changed to either D-Ala-D-Lactate (D-Ala-D-Lac) or D-Ala-D-Serine (D-Ala-D-Ser) by all forms of vancomycin resistance in enterococci (49). Modifications to the cell wall's structure, brought on by several genes, are the root cause of all forms of vancomycin resistance no matter how many types, the number and arrangement of these genes remain remarkably stable. The sensor gene vanS activates the regulator gene vanR in the presence of a glycopeptide, vanH facilitates the conversion of pyruvate to lactate when the gene complex is active while vanA utilizes lactate to create the alternative D-Ala-D-Lac end of the pentapeptide. Resistance can only exist if the pentapeptide's typical D-Ala-D-Ala terminus is no longer produced. The vanX and vanY genes, respectively, hydrolyze and stop the formation of the pentapeptides and cleave any pentapeptides that might still grow and provide a solution to this problem (49). VanS starts the process of dephosphorylating vanR when a glycopeptide is absent making the gene inactive. Reportedly vanA-type resistance was the first kind among others that induced highly resistant transposon Tn1546 and the other closely linked factors. The vanA ligase catalyzes the ester bond synthesis between D-Ala and D-Lac while vanH dehydrogenase converts pyruvate to D-Lac (30). As a result, the D-Ala-D-Ala dipeptide is changed into the D-Ala-D-Lac dipeptide which significantly reduces the molecule's affinity for glycopeptides (26).

Lipopolysaccharide (LPS) helps Gram-negative bacteria maintain a strong permeability barrier in their outer membrane, blocking the introduction of toxic substances like antibiotics. The important gene *cdsA*, which is involved in the transformation of phosphatidic acid into CDP-diacylglycerol during phospholipid biosynthesis, was the target of all seven suppressors that were examined. These *cdsA* mutations lead to a partial functional impairment as phosphatidic acid accumulation. The fact that these *cdsA* mutations provide a general rise in vancomycin resistance, even in a wild-type cell, means that this suppression is not limited to mutations that lead to abnormalities in outer membrane biogenesis. Researchers demonstrated that the accumulation of phosphatidic acid by methods other than *cdsA* mutations also enhances resistance to vancomycin using genetics and quadrupole time of flight (Q-TOF) liquid chromatography-mass spectrometry (LC-MS) (50). The authors hypothesize that elevated amounts of phosphatidic acid alter the outer membrane's physical characteristics to prevent vancomycin from entering the periplasm and reaching its target, an intermediate needed for the production of the peptidoglycan cell wall (50). Like zoonotic bacteria, resistant bacteria from food animals' intestinal flora contaminate animal corpses after being killed and then pass down the food chain to infect humans' intestines (51). It is possible to determine the prevalence of resistance in various populations and to identify a potential transmission of resistant bacteria from animals to individuals, and vice versa, by examining the prevalence of resistance of specific indicator organisms like *E. coli* and enterococci in the intestinal tract of various populations of animals and humans.

## The stance of the centers for disease control and prevention on the detection of VRSA

The samples processed for isolation and identification of *S. aureus* may grow on a mannitol salt agar or, alternatively, an MRSA chromogenic agar. Whatever method of identification is applied, the obtained *S. aureus* is required to evaluate against vancomycin antibiotic, and, if minimum inhibitory concentration (MIC) is found to be ≥4 μg/mL, it ought to be reported to health departments as reportable isolates. The MIC of vancomycin against *S. aureus* of ≥8 μg/mL must be sent to CDC for confirmation of VRSA. All the *S. aureus* isolates showing MIC ≥16 μg/mL against vancomycin are requested by the CDC for further characterization of VRSA precursor organisms. Upon confirmation by the CDC, this is shared with public health partners (52).

The guidelines for declaring different strains of *S. aureus* with respect to vancomycin are as follows:

1. VSSA (*S. aureus* sensitive to vancomycin) presenting MIC ≤ 2 μg/mL.
2. VRSA (*S. aureus* resistant to vancomycin) presenting MIC ≥ 16μg/mL.
3. VISA (*S. aureus* intermediate susceptible to vancomycin) presenting MIC 4-8 μg/mL.

VRSA was identified in 2002 in a patient hospitalized for a catheter-related infection. The MIC was noted >32 μg/mL and was related to VRE while molecular analysis showed a vanA positive gene of the Van cluster (53). Vancomycin resistance mechanisms are discussed in the following section, including intrinsic resistance seen in Gram-negative bacteria. Although recently some antibiotic alternatives like bacteriophages are being investigated against potential pathogens (54).

### Guidelines for VRSA

According to Hospital Infection Control Practices Advisory Committee (HICPAC), hospitals should develop their institution-specific protocols while the focus should remain as follows (55).

- The clinicians ought to prescribe vancomycin keeping in view the circumstances and consequences.
- Hospital staff should be aware of the vancomycin resistance.
- Detection and prompt reporting of vancomycin resistance in microorganisms from laboratories and hospitals should be early and prompt.
- An appropriate implementation plan for the control of horizontal infection (person to person) of VRE ought to launch.

The CDC suggests decolonizing patients from VRSA to avoid the development of clinical signs and the further spread of resistance. The colonization of VRSA in persons is defined as the presence of VRSA in or on a person but has no development of any clinical signs. The persons may simultaneously colonize with VRSA infection e.g., wound infection, and colonization of VRSA through the colonization of nares. Here it is important to decolonize (reducing the burden of pathogen/organism by eradication strategy). There are approaches suggested for this and among such is decreasing the reservoir of vancomycin-resistant *S. aureus* (VRSA). Certain factors like the disease situation and status of the immune response, capacity of the person to tolerate dose regimen, and risk of transmission to others make the baseline for decolonization. Such a decision further relies on the consultation of the patient's physician and public authorities (52).

## Public health concerns

Methicillin-resistant *S. aureus* is not only a recognized source of infection in hospitals (56) but also outside healthcare setups. The excessive use of vancomycin to treat MRSA increases the selective pressure of vancomycin in the population, leading to higher VISAs and VRSA strains (57). The introduction of food animals into Vancomycin-resistant Enterococci production systems was mostly linked to the widespread use of sub-treatment avoparcin to promote the growth of animals in Europe and other countries since the mid-1990s. Avoparcin was prohibited as a growth promoter in the EU in 1997 as described in Commission Directive 97/6/EC. VRE-related human illnesses and outbreaks, as well as the frequency of VRE in asymptomatic human carriers, have grown throughout the EU since 1999. The prohibition of disinfectant growth promoters may have played an important role in reducing VRE carriers in animals and glycopeptide-resistant Enterococci species. Despite the greater frequency of VRE carriers in animals, fewer nosocomial VRE infections were noted in Europe than in the United States even though avoparcin was never prescribed to North American animals. A decade or two ago, people found it difficult to explain food-borne infections except in a few cases of similar VREs identified from humans and animals which were believed to be spread from animal contact rather than meat intake. Human infections with animal VRE stress resulted in temporary colonization. However, evidence of various molecular typing techniques brought forward a strong correlation between VREs or genetic determinants in animals and humans. These links between animals and human VRE carriers have not been proven to be causal but show that there are several reverse transmissions from wild animals and plants (57).

## Concerns about food safety

Antibiotics were initially licensed to be used as growth promoters, while the abrupt ban on their use has compromised health due to critical food animal diseases. On the other hand, this ban has resulted in a reduction of resistance to pathogens of animals and human origin thus enhancing animal health and safety. According to a recent report by Denmark's National Department of Poultry Production, the broiler industry has been “plagued by leg and skin problems” since the late 1990s which could have jeopardized the broiler industry's wellbeing (58).

Food safety concerns about antimicrobial resistance were increased after the ban was implemented. There was a rise in infections which were earlier controlled by the use of medicine in the feed. The utilization of therapeutics in food-producing animals was increased in various parts of Europe. Sales of therapeutic antimicrobials grew from 383 tons in 1999 to 437 tons in 2000 after the EU imposed a ban on growth promoters in 1999. This was due to an increase in tetracycline sales of 36 tons, trimethoprim/sulphonamides sales of 12 tons, and macrolides sales of 12 tons. The use of medicines in pigs increased by 7 tons and use in the poultry sector increased by 13 tons. More than one species of animals showed an increase in the use of medicine by 37 tons. The EU restriction in 1999, as well as the incidents of ailments such as porcine dermatitis, nephritis syndrome, and post-weaning multisystemic wasting syndrome, were factors in the pig business's expansion. Therapeutic antibiotic use in Denmark increased from 48 tons in 1996 to 94 tons in 2001. Tetracycline, which is primarily used in pigs, increased from 12.9 to 27.9 tons (a 116 percent increase) followed by macrolides and lincosamides (7.6 to 14.3 tons, an 88 percent increase), and aminoglycosides (7.1 to 11.9 tons, 68 percent). Experience in Sweden suggests this might be somewhat beneficial in the end, albeit at a greater financial cost. However, it is unknown whether this will apply to the rest of Europe where farming conditions differ from those in Scandinavia (59).

## Economic losses due to resistance

In Europe, 9.6 percent of nosocomial infections were caused by Enterococcus species while VRE nosocomial infections were very frequent in Intensive Care Units (ICU). Serious basic health issues including liver transplants, diabetes mellitus, or kidney failure are risk factors associated with VRE colonization and the subsequent development of nosocomial infections. Several investigations have shown that VRE bloodstream infections were common (BSI) and far more deadly than vancomycin-susceptible enterococci BSI (VSE) (60). VRE infections necessitate higher invasive procedures, increased use of antibiotics, and longer hospitalization that led to both increased hospital expenditure and disease risk. In the United States, hospital expenditure for BSI-like VRE infections ranged from $9,949 to $77,558, as per data gathered from a university-based teaching hospital. Another study conducted in the United States discovered that the expenditure associated with VRE-related surgical site infections (SSI) was approximately $12,766. Despite the expense associated with these preventive measures, VRE screening in elevated areas and VRE client isolation also are well-established in many institutions. A study reported the hospitalization expense of VRE-infected patients was more than VSE-infected individuals. This could be because of factors associated with VRE patients' expenses than VSE patients' expenses. The economic impact of VRE infections in hospitalized patients is now being investigated in the United States and other countries.

## Conclusion

Vancomycin resistance has been found as an emerging issue that carries a wider range of resistant genes collectively called the “Van cluster”. These genes are found among a wider range of microbes that finally share these among each other. The transfer of resistance is extensive among animals, humans, and the environment with the full potential of zoonosis and reverse zoonosis. Significant economic damage is possible and food safety, food security, farm animal health, and public health are at risk. The adoption of a set of guidelines from the Centers for Diseases Controls and Prevention (CDC) ought to be implemented with an active reporting system. However, further protocols are required to control this resistance by developing coordinated but comprehensive therapeutic and preventive strategic plans.

## Author contributions

AIA wrote the manuscript. AFA revised the manuscript. All authors contributed to the article and approved the submitted version.

## Conflict of interest

The authors declare that the research was conducted in the absence of any commercial or financial relationships that could be construed as a potential conflict of interest.

## Publisher's note

All claims expressed in this article are solely those of the authors and do not necessarily represent those of their affiliated organizations, or those of the publisher, the editors and the reviewers. Any product that may be evaluated in this article, or claim that may be made by its manufacturer, is not guaranteed or endorsed by the publisher.
